# Identification of a Non-Invasive Urinary Exosomal Biomarker for Diabetic Nephropathy Using Data-Independent Acquisition Proteomics

**DOI:** 10.3390/ijms241713560

**Published:** 2023-09-01

**Authors:** Xiaonan Ding, Dong Zhang, Qinqin Ren, Yilan Hu, Jifeng Wang, Jing Hao, Haoran Wang, Xiaolin Zhao, Xiaochen Wang, Chenwen Song, Junxia Du, Fuquan Yang, Hanyu Zhu

**Affiliations:** 1 Department of Nephrology, First Medical Center of Chinese People’s Liberation Army General Hospital, Nephrology Institute of the Chinese People’s Liberation Army, National Key Laboratory of Kidney Diseases, National Clinical Research Center for Kidney Diseases, Beijing Key Laboratory of Kidney Disease Research, Beijing 100853, China; dingxiaonan99@163.com (X.D.); zhangdong1301@126.com (D.Z.);; 2 Medical School of Chinese People’s Liberation Army, Beijing 100853, China; 3 Laboratory of Proteomics, Institute of Biophysics, Chinese Academy of Sciences, Beijing 100101, China

**Keywords:** diabetic nephropathy, non-diabetic renal disease, urine exosomes, proteomics

## Abstract

Diabetic nephropathy (DN), as the one of most common complications of diabetes, is generally diagnosed based on a longstanding duration, albuminuria, and decreased kidney function. Some patients with the comorbidities of diabetes and other primary renal diseases have similar clinical features to DN, which is defined as non-diabetic renal disease (NDRD). It is necessary to distinguish between DN and NDRD, considering they differ in their pathological characteristics, treatment regimes, and prognosis. Renal biopsy provides a gold standard; however, it is difficult for this to be conducted in all patients. Therefore, it is necessary to discover non-invasive biomarkers that can distinguish between DN and NDRD. In this research, the urinary exosomes were isolated from the midstream morning urine based on ultracentrifugation combined with 0.22 μm membrane filtration. Data-independent acquisition-based quantitative proteomics were used to define the proteome profile of urinary exosomes from DN (n = 12) and NDRD (n = 15) patients diagnosed with renal biopsy and Type 2 diabetes mellitus (T2DM) patients without renal damage (n = 9), as well as healthy people (n = 12). In each sample, 3372 ± 722.1 proteins were identified on average. We isolated 371 urinary exosome proteins that were significantly and differentially expressed between DN and NDRD patients, and bioinformatic analysis revealed them to be mainly enriched in the immune and metabolic pathways. The use of least absolute shrinkage and selection operator (LASSO) logistic regression further identified phytanoyl-CoA dioxygenase domain containing 1 (PHYHD1) as the differential diagnostic biomarker, the efficacy of which was verified with another cohort including eight DN patients, five NDRD patients, seven T2DM patients, and nine healthy people. Additionally, a concentration above 1.203 μg/L was established for DN based on the ELISA method. Furthermore, of the 19 significantly different expressed urinary exosome proteins selected by using the protein–protein interaction network and LASSO logistic regression, 13 of them were significantly related to clinical indicators that could reflect the level of renal function and hyperglycemic management.

## 1. Introduction

The global prevalence of diabetes in 2019 was estimated to include 463 million people, with a significantly increased incidence that is projected to reach 700 million by 2045 [1]. Diabetic nephropathy (DN), as a serious microvascular complication, affects 20–40% of Type 1 diabetes mellitus (T1DM) and Type 2 diabetes mellitus (T2DM) patients [2]. In the developed world, DN is the leading cause of end-stage renal disease (ESRD), requiring dialysis or kidney transplantation and imposing a huge economic burden on society [3]. DN is generally a clinical diagnosis that is made based on a longstanding duration of diabetes, the presence of albuminuria, and/or a reduced estimated glomerular filtration rate (eGFR) in the absence of evidence for other primary causes of renal damage [2]. However, the clinical manifestations are not always typical, as some patients have comorbid diabetes and primary kidney disease, such as IgA nephropathy (IgAN), membranous nephropathy (MN), and focal segmental glomerulosclerosis (FSGS). Impaired kidney function can be caused by primary kidney disease and not diabetes, and this comorbid disease is defined as non-diabetic renal disease (NDRD) [4]. DN and NDRD differ in many aspects, including their pathological characteristics, therapeutic regimen, disease progression, and prognosis [5]. Therefore, it is important to accurately differentiate between the diagnosis of DN and NDRD. Some clinicians differentially diagnose DN and NDRD based on clinical experience, which could possibly be inaccurate, leading to a delay in the opportunity for timely treatment. The gold standard for differentially diagnosing DN and NDRD is a renal biopsy, which is an invasive, time-consuming, and expensive procedure that requires high technical proficiency [2,6]. Therefore, there is an urgent need to find an effective non-invasive method for differentiating between DN and NDRD.

Urine has great advantages over other biological fluids produced directly by the kidney; as it is collected non-invasively, it is viewed as a valuable diagnostic medium. To date, numerous studies have been conducted to identify a biomarker, and urine exosomes, as one of the components of urine, can identify proteins not found in the whole urine, fueling an opportunity to find a new biomarker for DN and NDRD [7]. Exosomes are defined as a heterogeneous group of spherical bilayered proteolipid structures, 50–150 nm in diameter, that originate from the endosomal system of basically all cell types and are released by plasma membrane fusion [8]. In recent years, studies on exosomes have burgeoned, including various constituents such as proteins, amino acids, nucleic acids, lipids, and metabolites, which could reveal valuable pathophysiological information [9,10]. Additionally, exosomes are speculated to remove and uptake constituents from the cells to maintain homeostasis, control the sensitivity of cell death, remodel the microenvironment, and participate in immune responses and infection; this may not only regulate cellular crosstalk and intercellular communications but may also reflect the physiological state and pathological conditions of the originating cells [11,12,13,14]. They can be detected in a variety of biological fluids such as blood, urine, cerebrospinal fluid, etc. [15]. In previous studies, several urine exosome proteins were found to be significantly changed in DN patients, such as alpha-1-microglobulin/bikunin precursor (AMBP), full-length megalin, and dipeptidyl peptidase-4(DPP-4) [16,17,18,19,20,21]. However, the difference in the urinary exosomal proteomic profile between DN and NDRD is still unknown.

The present study defined the proteome profile of urine exosomes in patients diagnosed with renal puncture in depth and aimed to (1) compare the proteins of urinary exosomes in different subjects, (2) enrich the putative biochemical functions of significantly expressed proteins, (3) screen potential diagnostic biomarkers to distinguish between DN and NDRD, (4) verify the efficacy of the non-invasive differential diagnostic biomarkers, and (5) analyze the correlation between the proteins of the urinary exosomes and renal function. For this, urinary exosomes were isolated using ultracentrifugation combined with 0.22 μm filtration, and proteomic profiles were acquired by using the data-independent acquisition mass spectrometry (DIA-MS) strategy.

## 2. Results

### 2.1. Characterization of the Exosomes

**Figure 1:**
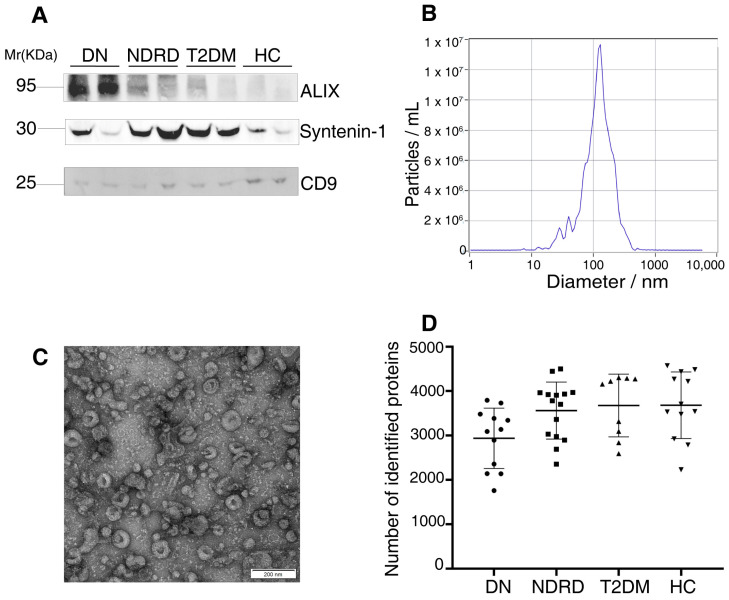
(**A**) Western blot analysis showing that isolated urinary exosomes were positive for at least two exosome markers. (**B**) Nanoparticle tracking analysis indicated that the particle size was mainly distributed at 127.2 nm. (**C**) The size and structure of the exosome were confirmed by transmission electron microscopy. Magnification: 98,000×. (**D**) The numbers of identified proteins showed insignificant differences among the groups. DN, diabetic nephropathy; NDRD, non-diabetic renal disease; T2DM, Type 2 diabetes mellitus; HC, healthy controls.

The Western blot analysis revealed that the urine exosomes had at least two positives for ALIX, Syntenin-1, and CD9 (Figure 1A) [22]. The NTA results of randomly selected samples showed that the particles’ size was mainly distributed at 127.2 nm, which is consistent with the typical sizes of exosomes in the range from 40 to 150 nm (Figure 1B). TEM showed pellets containing spherical membranous vesicles ranging in size from 40 to 150 nm with less coprecipitation (Figure 1C).

### 2.2. Proteomic Analysis

To identify the protein of urine exosomes as a differential diagnostic biomarker for DN and NDRD, 48 first morning urine samples were collected midstream from 12 patients with DN, 15 patients with NDRD, 9 patients with T2DM, and 12 healthy subjects. After the urine exosome isolation process mentioned above, the samples were individually profiled by DIA-MS data acquisition. Table 1 summarizes the basic demographic and clinical characteristics of the subjects.

**Figure 2:**
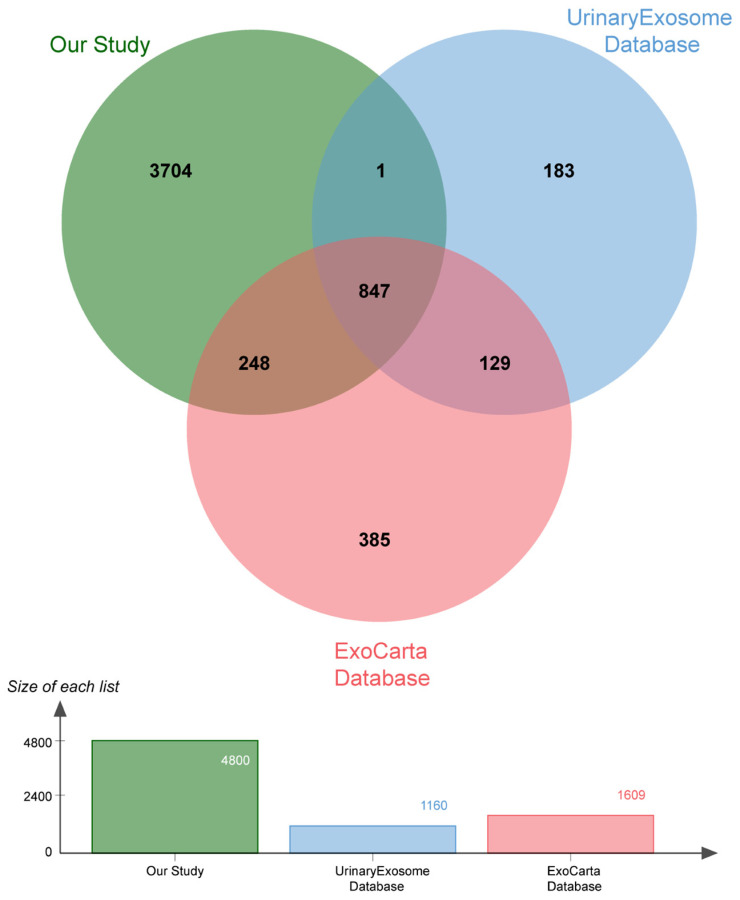
Venn diagram showing the number of urinary exosome proteins compared with the ExoCarta and UrinaryExosome databases.

**Figure 3:**
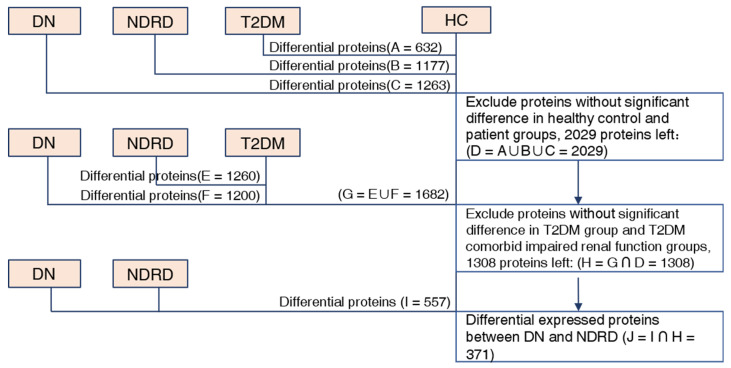
Flowchart of screening the differential expressed proteins between DN and NDRD. DN, diabetic nephropathy; NDRD, non-diabetic renal disease; T2DM, Type 2 diabetes mellitus; HC, healthy controls.

**Figure 4:**
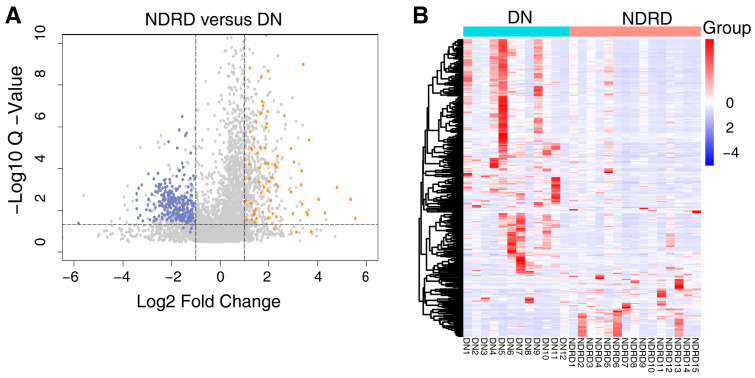
(**A**) Volcano plot of 371 differentially expressed proteins between DN and NDRD, with orange indicating upregulated expression, blue indicating downregulated expression compared with DN and grey indicating there are no significance between DN and NDRD. (**B**) Heat map of all 371 potential biomarker proteins were clustered by DN and NDRD. In a heat map, each row represents a protein, and each column corresponds to each sample. The normalized Z score of protein abundance is depicted by a pseudocolor scale, with red indicating upregulated expression, white indicating equal expression, and blue indicating downregulated expression compared with the values of each protein, whereas the tree dendrogram displays the results of an unsupervised hierarchical clustering analysis placing similar proteome profile values near each other. The dendrogram and heat map demonstrate the ability of these proteins to distinguish between DN patients and NDRD patients. DN, diabetic nephropathy; NDRD, non-diabetic renal disease.

The protein composition of the urine exosomes from 48 subjects was individually determined, and an average of 3372 ± 722.1 proteins for each participant was identified. The number of identified proteins showed no significant differences among the groups (Figure 1D). In total, 4800 proteins were identified in all subjects. We compared our data with ExoCarta database (http://www.exocarta.org) accessed on 16 August 2023, which contains 1609 urinary exosome proteins. However, 1095 proteins have been reported and 3705 were not included. When compared with another urinary exosomal dataset (https://esbl.nhlbi.nih.gov/UrinaryExosomes/) accessed on 16 August 2023, which included 1160 proteins identified from urine samples from healthy people, 848 proteins overlapped and 3952 proteins were identified in our study. What is interesting is these two databases overlapped by just 976 proteins. When combined with our study, 847 proteins were identified as common; the results are shown in Figure 2 and detailed data are shown in Supplementary Table S1 [23,24,25]. ∣Log_2_Fold Change∣ ≥ 1 and a Q value of <0.05 were used as the cutoff criteria to perform the differential expression analysis. The proteins of different groups were compared in pairs; Table 2 shows the number of upregulated and downregulated proteins. To screen out potential biomarkers that expressed a significant difference between DN and NDRD, we followed the process shown in Figure 3. First, we excluded the most commonly expressed urine exosome proteins in all subjects, meaning that 2029 significantly and differently expressed proteins entered the next step. After that, the urine exosome proteins of T2DM patients and patients with T2DM as a comorbidity with impaired renal function (DN and NDRD) were separately compared; the most commonly expressed proteins were excluded and were considered as changed by the pathological background of T2DM. This left 1308 proteins that were further screened, of which 371 proteins were shown to be significantly and differently expressed between DN and NDRD, where 291 proteins were significantly less abundant and 80 proteins were more abundant in NDRD patients compared with the DN group (Figure 4A). In addition, Figure 4B reveals that 371 proteins were significantly and differentially clustered between the DN and NDRD patients, and Table 3 shows the top 10 upregulated proteins and the top 10 downregulated proteins exhibiting the highest differences.

### 2.3. Bioinformatic Analysis of Differentially Expressed Proteins

**Figure 5:**
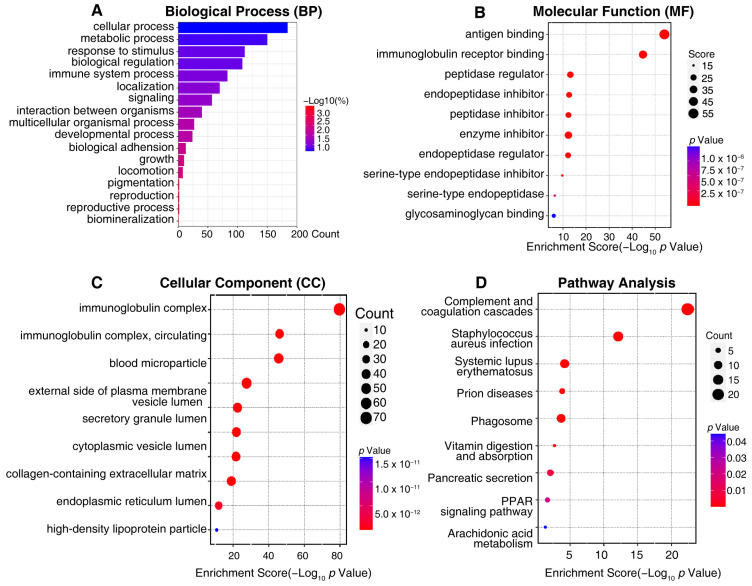
The visualized results of representative functional enrichment-based GO analysis of 371 potential biomarkers of a differential diagnosis between DN and NDRD patients. (**A**) Biological process analysis. (**B**) Molecular function analysis. (**C**) Cellular component analysis. The ordinate indicates the enrichment’s classification, the color gradient represents the *p*-value, and the size of the bubbles represents the number of counts. (**D**) In pathway analysis, the color of bubbles represents the adjusted P-value, and the size of the bubbles represents the number of counts.

To better understand the biological function of the differentially expressed urine exosome proteins, GO annotations and an enrichment analysis of 371 proteins were performed to elucidate the possible functional differences of the urine exosomes originating from DN and NDRD. An analysis was performed for three categories: cellular components, biological processes, and molecular functions. Additionally, the results revealed that the most enriched biological processes in the 371 differently expressed urine exosomes proteins related to cellular processes, metabolic processes, responses to stimuli, biological regulation, immune system processes, localization, and signaling (Figure 5A). The molecular function analysis showed that the most enriched functions included antigen binding, immunoglobulin receptor binding, the peptidase regulator, the endopeptidase inhibitor, the peptidase inhibitor, the enzyme inhibitor, etc. (Figure 5B). Regarding the cellular components of differently expressed urine exosomes proteins, the immunoglobulin complex, blood microparticles, the external side of the plasma membrane, and vesicle lumens were defined (Figure 5C). KEGG enrichment analysis suggested that the differently expressed urine exosome proteins were mainly involved in complement and coagulation cascades, pancreatic secretion, the phagosome, vitamin digestion and absorption, the PPAR signaling pathway, and arachidonic acid metabolism (Figure 5D). Regarding the complement and coagulation cascade pathway, the significant urine exosome proteins involved included F12, AT3, F7, F9, F10, α2AP, CPB2, C1qs, and C5b-9, etc. (Supplementary Figure S1A), alongside Rabs, Rap1, CD38/157, amyA, and CPB in the urine exosomes, which were engaged in the pancreatic secretion pathway (Supplementary Figure S1B). In addition, Supplementary Figure S1C shows vATPase, TUBB, CALR, calnexin, FcγR, CR3, αMβ2, iC3b, TSP, MPO, and gp91 in the phagosome pathway and FABP, Apo-AI, Apo-AII, Apo-CIII, LPL, and aP2 in the PPAR signaling pathway (Supplementary Figure S1D). The vitamin digestion, absorption, and arachidonic acid metabolism pathways are shown in Supplementary Figure S1E,F.

### 2.4. Screening for Potential Biomarkers

**Figure 6:**
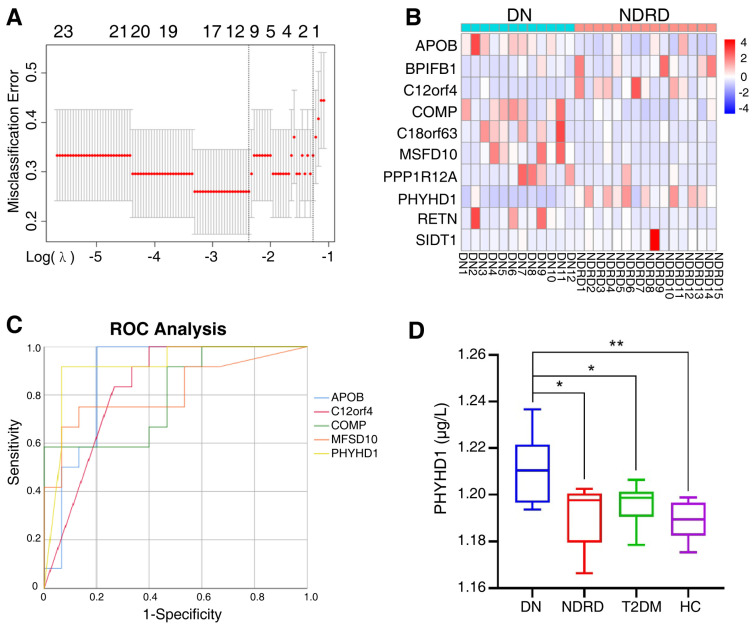
(**A**) The two dashed lines indicate λ.min and λ.1se, respectively, and the λ between these two values is considered to be appropriate; λ.1se builds the simplest model and uses a small number of proteins, while λ.min builds a model with higher accuracy and uses more proteins. (**B**) The 10 candidate proteins screened by the LASSO regression via the λ.min model, clustered in clustering heat maps for DN and NDRD. In a heat map, each row represents a protein and each column corresponds to each sample. The normalized Z score of protein abundance is depicted by a pseudocolor scale, with red indicating upregulated expression, white indicating equal expression, and blue indicating downregulated expression compared with the values of each protein. (**C**) ROC analysis constructed for the diagnostic accuracy of selected urine exosome proteins determined to have an AUC of 0.8 or over. ROC, receiver operating characteristic, AUC, the area under the curve. (**D**) ELISA quantified the PHYHD1 of urine exosomes in the DN, NDRD, T2DM, and HC groups; * *p* < 0.05, ** *p* < 0.01. DN, diabetic nephropathy; NDRD, non-diabetic renal disease; T2DM, Type 2 diabetes mellitus; HC, healthy control.

After primary screening, 371 proteins were significantly and differentially expressed in the urine exosomes of DN and NDRD patients as potential biomarkers. To further identify significantly and differentially expressed proteins and to compress the high-dimensional data, LASSO regression was used to obtain the regression coefficients based on the penalty function approach (Figure 6A), in which λ.1se was 0.2828 while λ.min was 0.093. Additionally, the λ.1se model included only one protein (PHYHD1), and the λ.min model included 12 proteins, among which PRSS1 was the trypsin used in the solution for enzyme digestion and KRT33B was associated with the reproductive system; therefore, we excluded these two proteins from the following analysis. The clustering heat map of 10 candidate proteins between DN and NDRD patients is shown in Figure 6B.

In addition, the ROC method was used to further analyze the candidate proteins. Figure 6C revealed that the area under the curve (AUC) of the urine exosome proteins was 0.8 or over, including PHYHD1 (AUC = 0.919), APOB (AUC = 0.878), C12orf4 (AUC = 0.828), MFSD10 (AUC = 0.814), and COMP (AUC = 0.800). Additionally, the AUC data of all 10 candidate proteins are shown in Supplementary Table S2.

To investigate the diagnostic accuracy of multiple urine exosome proteins combined, a univariate logistic regression analysis was performed on the remaining 10 candidate proteins. The results of the univariate logistic regression analysis are listed in Supplementary Table S3, where APOB (*p* = 0.015), COMP (*p* = 0.025), C12orf4 (*p* = 0.043), PHYHD1 (*p* = 0.009), and SIDT1(*p* = 0.015) were shown to be significant; these five selected variables were used for the multivariate logistic regression analysis. The results showed that the significance of the omnibus test was <0.01, and the significance of the Hosmer–Lemeshow test was 0.516, indicating the goodness of fit. Additionally, only the PHYHD1 protein had significance, which was consistent with the results showing only PHYHD1 to have variance for the λ.1se model in the LASSO regression analysis, and PHYHD1 had the top AUC in the ROC analysis. Therefore, PHYHD1 was considered to be the urine exosome protein to be the biomarker for a differential diagnosis between DN and NDRD.

### 2.5. Validation of Biomarker

To verify the efficiency of PHYHD1 as the biomarker for differential diagnosis, urine exosomes were isolated from eight patients with DN, five patients with NDRD, seven patients with T2DM, and nine healthy people; the characteristics of the 29 subjects are shown in Table 4. Then the previously identified urine exosome biomarker PHYHD1 was tested using an ELISA kit (Abbexa Ltd., Milton, UK), which showed the concentration of PHYHD1 was significantly and differently expressed between DN and NDRD, between DN and T2DM, as well as between DN and healthy individuals (Figure 6D). ROC curve analysis was used to determine the optimum (highest sum of sensitivity and specificity) concentration of PHYHD1 in the urine exosomes for the differential diagnosis between DN and NDRD. A concentration above 1.203 μg/L was established for DN with 100% sensitivity and 75% specificity (AUC = 0.850).

### 2.6. Clinical Correlation

**Figure 7:**
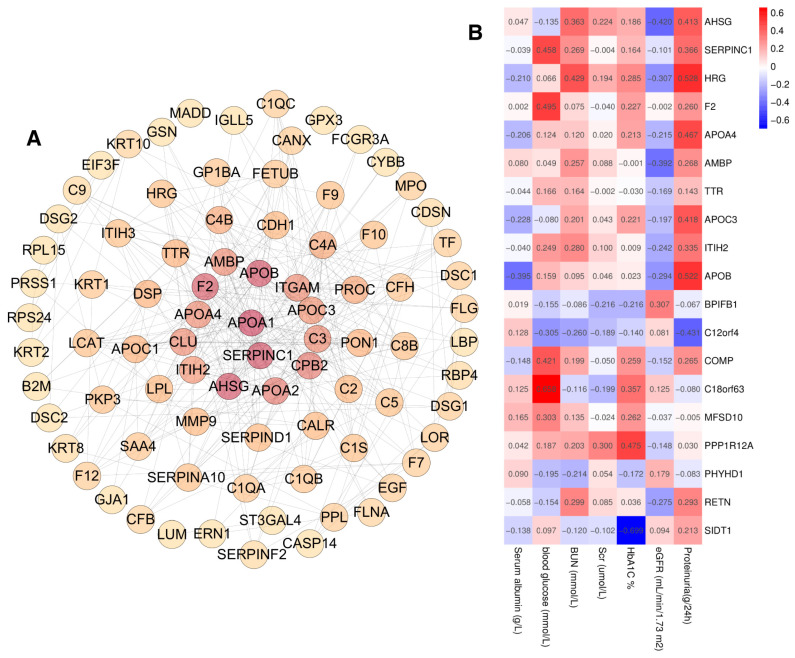
(**A**) Protein–protein interaction analysis of 371 proteins with significantly different expressions of DN and NDRD, the enrichment score is depicted by a pseudo color scale and size, (**B**) Pearson’s correlation analysis of 19 proteins and clinical parameters.

To investigate the correlation with the urinary exosome proteins and the clinical indicators, first, PPI analysis was performed on 371 proteins with significantly different expression between DN and NDRD, and the minimum interaction score was set to 0.70, resulting in a total of 294 nodes and a PPI enrichment of *p* < 0.01 (Figure 7A). After that, hub objects and subnetworks were identified using the maximal clique centrality (MCC) of the cytoHubba module in Cytoscape 3.8.2., and the top 10 key proteins and the proteins selected via the LASSO analysis (19 proteins in total) were further analyzed for their association with clinical parameters using Pearson’s correlation analysis (Figure 7B). On the basis of the 48 participants in the identification group, we found that proteinuria correlated positively with the levels of AHSG, APOA4, APOB, APOC3, and HRG, and correlated negatively with C12orf4. Additionally, the estimated glomerular filtration rate (eGFR) correlated negatively with AHSG and AMBP. Blood urea nitrogen (BUN) correlated positively with HRG. Serum albumin correlated negatively with APOB, while blood glucose correlated positively with the level of C18orf63, COMP, F2, and SERPINC1. Additionally, glycated hemoglobin (HbA1c) correlated positively with PPP1R12A and negatively with SIDT1, whereas there was no significant correlation for BPIFB1, ITIH2, MFSD10, PHYHD1, RETN, and TTR with the clinical parameters.

## 3. Discussion

The exosome is one of three categories of extracellular vesicles, which also include microvesicles (or ectosomes) and apoptotic bodies [26]. This classification is based on the difference in their biogenesis: exosomes (50–150 nm in diameter) originate from the endosomal system and plasma membrane fusion for their release, microvesicles (50–1000 nm in diameter) are directly shed from the plasma membrane, and apoptotic bodies (800–5000 nm in diameter) are generally shed from dying cells [27]. Existing isolation and purification approaches are based on the difference in size, density, and the immune precipitation of pellets, such as differential ultracentrifugation, ultrafiltration, size exclusion chromatography, and immunoaffinity capture [28]. However, the size and density of exosomes and microvesicles partially overlap, and the biomarkers are suboptimal for distinguishing between the two, as not all exosomes express the same classical membrane protein markers, nor are all established markers exclusive to the exosomes, as has been found for other subtypes of extracellular vesicles as well [29,30]. However, it is necessary to distinguish exosomes and microvesicles due to numerous studies which have indicated that exosomes and microvesicles exhibit different proteomic profiles and biological functions [31]. Therefore, detailed reports on the procedure of their isolation and characterization can help to define the limitations of the study and specify the heterogeneity. Additionally, there is no standard methodology that is applicable to all kinds of studies for the selection or combination of procedures depends upon downstream research [32]. For example, uromodulin, also known as the Tamm–Horsfall protein, is one of the most abundant proteins excreted into the urine, and the network of filamentary polymeric uromodulin could entrap the exosomes and cause them to coprecipitate, leading to a decreased yield [33,34]. Polymeric uromodulin can be depolymerized, and its ability to oligomerize is abolished in order to release the exosomes by adding dithiothreitol to break the disulfide bridges; however, dithiothreitol can also break the disulfide bond in the proteins of exosomes, which probably changes the proteomic profile to prevent the accuracy of further analysis, though it might be acceptable in the analysis of microRNA [34]. Moreover, this technique of isolation is not only hard to define and purify to obtain the specific subgroup of extracellular vesicles but also limits its application in clinical practice due to the time consumed, the labor intensity, and the expensive equipment [28].

Urine exosomes originate from various sources, including the kidneys, bladder, genitourinary tract cells, immune cells, etc. In addition, under pathological conditions, damaged podocytes and injured basement membranes could partly allow the exosomes to enter the urine from the circulatory system [17]. Therefore, the various sources of urine exosomes indicate that they may contain comprehensive pathophysiological information but also bring challenges in that it is difficult to figure out in the originating cell of the exosomes to further identify the potential therapeutic targets [9]. As for this study, although we identified phytanoyl-CoA dioxygenase domain containing 1 (PHYHD1), which could be a potential diagnostic biomarker, and conducted an enrichment analysis of significant and differentially expressed proteins, the cellular source is still unknown, which prevents a further analysis of the pathological process.

The differential diagnostic biomarker, the urinary exosome PHYHD1, is related to the peroxisomal lipid metabolism pathway. PHYHD1 exists in three isoforms and PHYHD1A, but likely not the PHYHD1B/C isoforms, catalyzes the conversion of 2-oxoglutarate to succinate and CO_2_ in an iron-dependent manner; however, it is not directly involved in phytanoyl Coenzyme A metabolism [35]. Ferroptosis is an iron-dependent and non-apoptotic cell death driven by overreactive lipid peroxidation culminating in irreversible plasma membrane damage, and exosomes have been found that can drive resistance to ferroptosis [36,37,38,39]. Under diabetic conditions, lipid peroxidation is significantly increased, and ferroptosis is also involved in kidney tubular cells’ death [40]. However, it is still unknown whether PHYHD1, as a functional Fe and 2OG-dependent oxygenase, plays a role in this process. In addition, PHYHD1, as a PHYH-like homolog, is also considerably expressed in T cells and B cells, and was upregulated in T cells after stimulation, which was related to T cell differentiation and/or the function of effector T cells [41]. We found that PHYHD1 contributed to the differential diagnosis between DN and NDRD patients in a performant way; however, it showed no significant relation to kidney function and blood sugar. It is rational that commonly clinical indicators such as eGFR and proteinuria could just indicate the kidney’s function but not the specific category of kidney disease.

Although the amounts of protein were not considered as a biomarker to differentially diagnose DN and NDRD, several of them still showed significant differences, such as C12orf4. The top transcription factor binding sites in the C12orf4 gene promoter were AREB6, E2F, PPAR-γ, and ROR α2. AREB6, also named ZEB1, δ-EF1, or TCF8, is one of the ZEB family of zinc finger transcription factors that induce epithelial to mesenchymal transition (EMT). E-cadherin is a major target gene of these transcriptional repressors, and this downregulation is considered a hallmark of EMT [42]. Previous studies have verified that under the circumstance of diabetes, downregulated miR-192 in the proximal tubular cells enhances the expression of ZEB1, thereby activating the TGF-β-mediated downregulation of E-cadherin [43,44]. In this study, we found that C12orf4 was significantly under-expressed in the urine exosomes of DN patients and was negatively related to proteinuria. Therefore, downregulated C12orf4 in the urinary exosomes suggests that the downregulation of ZEB1 promotes fibrogenesis in DN patients, which could partly explain the poor prognosis of DN compared with NDRD.

Our data revealed that the urinary exosomal ApoB was significantly upregulated in DN patients and was also related to proteinuria, serum albumin, and BUN. A recent study considered ApoB to be a contaminant in isolated exosomes from the blood, whereas, in this study, we collected the exosomes from urine, where ApoB barely exists [28,45]. It is interesting that the presence of ApoB in urinary extracellular vesicles is a biomarker for malignant bladder cancer [46]. As for SIDT1, which negatively correlated with HbA1c, it localizes to the endolysosomal compartment expressed in the lymphoid lineage and at the crossroad between the IFN-I and the proinflammatory pathways [47,48]. The alpha(2)-Heremans-Schmid glycoprotein (AHSG) was shown to be positively related to proteinuria and negatively to eGFR, which is in accordance with previous studies that found high AHSG plasma levels to be associated with insulin resistance in humans, and that the attenuation of AHSG could improve insulin resistance [49,50]. Peroxisome proliferator-activated receptors (PPARs) modulate several biological processes that are perturbed, including insulin resistance, glucose intolerance, obesity, dyslipidemia, hypertension, atherosclerosis, albuminuria, and inflammation [51,52]. Synthetic thiazolidinediones (TZDs) and PPAR-γ agonists, including rosiglitazone and pioglitazone, could effectively improve insulin sensitivity and lower the blood glucose level in patients with Type 2 diabetes [53]. In this study, we found that several urinary exosome proteins enriched in the PPAR pathway were consistent with the importance of this in T2DM and DN; however, how the urine exosomes participate in pathological and pharmacological processes is still unknown.

### Study Limitations

Though our assays’ results were promising, this was a single-center study. The results merit validation in a larger cohort. Due to the fact that renally impaired patients with T2DM confirmed by renal biopsy are particularly valuable, the limited number of cases obstructed further analysis of the severity of pathological changes. Additionally, the isolation procedure of the urinary exosome is still labor-intensive and time-consuming. If the status quo of exosome isolation and purification technology can be improved, then the clinical application will be more widespread. There might be some mechanisms of urinary exosome proteins that we do not know and have not yet discovered, which need further work to explore.

## 4. Materials and Methods

### 4.1. Description of the Cohort

Between May 2017 and June 2021, 77 eligible subjects were enrolled in this study. Midstream morning urine samples were collected from 21 healthy people, 20 resident patients diagnosed with DN, 20 resident patients diagnosed with NDRD, and 16 resident T2DM patients diagnosed not to have kidney damage.

The 21 healthy individuals were recruited during annual physical examinations, including routine urinalysis, a complete blood count, blood chemistry, tumor antigen tests, a chest X-ray, and abdominal ultrasonography. None of them showed any evidence of malignancy in all these tests. All enrolled subjects, except the healthy individuals, fulfilled the American Diabetes Association (ADA) criteria for T2DM and, on the basis of their clinical manifestations and endocrine laboratory measurements, the T2DM group also had a urinary albumin-to-creatinine ratio (UACR) of <30 mg/g and normal serum creatinine [54]. The 20 DN patients and 20 NDRD patients with longstanding T2DM, who volunteered to undergo a kidney biopsy, were recruited after percutaneous renal puncture. Histopathological confirmation was performed by two pathologists independently, according to the Renal Pathology Society’s classification system [2,55]. Patients meeting the following criteria were excluded: (1) those with an incomplete or unclear medical history, (2) confirmed renal disease before the diagnosis of T2DM, (3) familial or hereditary nephropathy, (4) comorbid urinary tract infections, (5) malignant neoplasms, (6) immune system diseases, or (7) pregnancy, and (8) those who had entered end-stage renal disease [56].

### 4.2. Urine Exosomes Isolation

**Figure 8:**
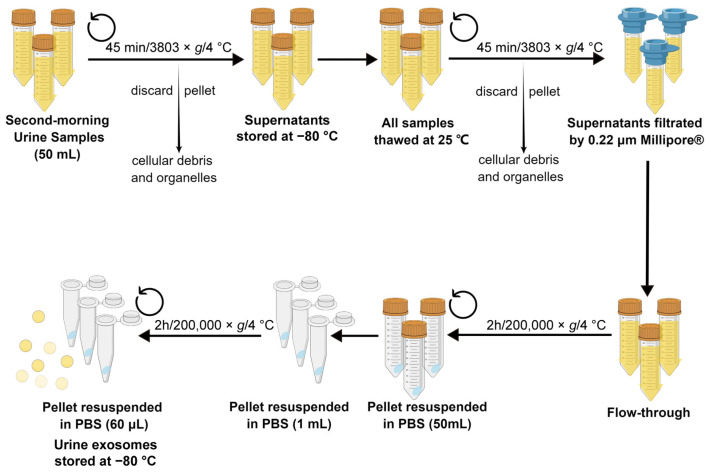
Flowchart showing the procedure of isolating urine exosomes. The drawing was created using www.figdraw.com software 2.0 (accessed on 20 June 2023). PBS: phosphate-buffered saline.

A flowchart of the isolation of urine exosomes is shown in Figure 8. Firstly, before the renal biopsy was conducted, midstream first morning urine samples (50 mL each) were obtained from each eligible participant. Samples without protease inhibitor cocktails were immediately centrifuged (3803× *g* at 4 °C for 45 min) to remove cellular debris and organelles. The obtained supernatants were stored at −80 °C for exosome isolation [8]. After the sample collection phase, all samples were thawed at 25 °C and centrifuged again; after that, the obtained supernatants were filtrated by using a 0.22 μm polyethersulfone filter sterilization device and the flow-through was centrifugated three times (200,000× *g* at 4 °C for 2 h) to obtain sedimental exosome-containing pellets. The pellets were resuspended in 50 mL of phosphate-buffered saline (PBS) and stored at −80 °C [21]. The isolation of the urine exosomes of all samples was performed by the first author to minimize experimental error and the batch effect. Details on the extraction and purification of exosomes are shown in the Supplementary Materials.

### 4.3. Characterization of the Exosomes

The isolated urine exosomes were characterized by following the guidelines for urinary extracellular vesicles of the Urine Task Force of the International Society for Extracellular Vesicles (2021) [8].

Therefore, urine exosomes were characterized in terms of (1) specific and/or abundant protein markers (ALIX, Syntein-1, CD9); (2) the size distribution of the final pellet, which was evaluated by using nanoparticle tracking analysis (NTA); and (3) morphological characteristics, which were assessed using transmission electron microscopy (TEM).

Western blot analysis was used to assess the protein biomarkers of the urine exosomes, where the final obtained pellets were separated via SDS-PAGE (12% Bis-Tris) and then transferred to a PVDF membrane. The antibodies were anti-ALIX (Santa Cruz, Dallas, TX, USA, sc-53540), anti-Syntenin-1 (Abcam, Cambridge, UK, ab19903), and anti-CD 9 (Abcam, ab236630) with HRP-conjugated secondary antibodies of anti-rabbit (Beyotime, A0208) and anti-mouse (Beyotime, Shanghai, China, A0216) [8]. The ChemiDoc Touch Imaging System and Image Lab software (Bio-Rad 6.1) were used to detect and quantify the immunoreactive bands.

NTA was performed to evaluate the size distribution of the final pellets. Urine exosome aliquots were diluted 50-fold in PBS, and the size of the exosomes was determined by using a Particle Metrix Zeta VIEW nanoparticle tracking analyzer (Particle Metrixc, Meerbusch, Germany), which tracked the scattered light of each particle and obtained a trajectory of Brownian motion for 60 s. This process was repeated three times before the concentration and size of the individual vesicles were calculated through the Stokes–Einstein equation [57].

TEM was used to visualize the morphology. Exosome-containing pellets were thawed at 25 °C and mixed with an equal volume of 4% paraformaldehyde. Resuspended pellets (5 μL) were deposited on Formvar carbon-coated copper electron microscopy grids for 1 min and were gently absorbed with filter paper. After transferring the grid twice to a 10 μL drop of 4% uranyl acetate, the excess fluid was gently removed by using filter paper. We left a drop of uranyl acetate behind to obtain the essential thickness of the film. After drying, the negatively stained exosomes were observed under an electron microscope at 80 kV [58].

### 4.4. Proteomic Analyses

After protein digestion based on the quantification of the protein of urine exosomes analyzed by using the bicinchoninic acid assay (BCA), a solution containing 10 μg of protein by volume per sample was uniformly dissolved to 15 μL with 8 M urea/100 mM NH_4_HCO_3_. Additionally, 0.3 μL of 0.5 M DTT was added to the sample for deoxidation at 37 °C for 2 h. After that, the reacting system was alkylated with 0.6 μL of 0.5 M iodoacetamide (IAM), which was protected from the light for 45 min at 25 °C. After being diluted with 45 μL of 50 mM NH_4_HCO_3_, 1 μL of 0.5 μg/μL trypsin was added to each sample for digestion at 37 °C for 16 h. At the end of the reaction, 10% formic acid (FA) was added to the sample for termination.

DIA-MS Data Acquisition—All nano HPLC-MS/MS experiments were performed on an Orbitrap Eclipse (Thermo Scientific, Waltham, MA, USA) equipped with an Easy n-LC 1200 HPLC system (Thermo Scientific). The peptides were loaded onto a 100 μm i.d. × 2 cm fused silica trap column packed in-house with reverse-phase C18 (Reprosil-Pur C18 AQ, 5 μm, Dr. Maisch GmbH, Ammerbuch, Germany) and were then separated on a 75 μm i.d. × 25 cm C18 column packed with reverse-phase C18 (Reprosil-Pur C18 AQ, 1.9 μm, Dr. Maisch GmbH). The peptides that were bound on the column were eluted across a 10^3^ min gradient. Solvent A consisted of 0.1% formic acid in a water solution, and Solvent B consisted of 80% acetonitrile and 0.1% formic acid. The segmented gradient was 4–11% B for 4 min, 11–21% B for 28 min, 21–30% B for 29 min, 30–42% B for 27 min, 42–99% B for 5 min, and 99% B for 10 min at a flow rate of 300 nL/min.

The MS analysis was performed with an Orbitrap Eclipse mass spectrometer (Thermo Scientific). In the data-independent acquisition mode, MS data were acquired at a high resolution of 120,000 (*m*/*z* 200) across the mass range of 400–1210 *m*/*z*. The target value was 4.00 × 10^5^ with a maximum injection time of 50 ms. One full scan was followed by 40 windows with an isolation width of 16 *m/z* for fragmentation in the ion routing multipole with an HCD normalized collision energy of 30%. MS/MS spectra were acquired at a resolution of 30,000 at *m*/*z* 200 across the mass range of 200–2000 *m*/*z*. The target value was 4.00 × 10^5^, with a maximum injection time of 50 ms. For the nanoelectrospray ion source’s settings, the spray voltage was 2.0 kV; if there was no sheath, there was no gas flow, and the heated capillary’s temperature was 320 °C.

DIA Data Analysis—Raw DIA data from the Orbitrap Eclipse were analyzed using Spectronaut version 14 (Biognosys, Schlieren, Switzerland) in the “DirectDIA” mode for the identification and quantification of proteins. The Uniprot human protein database (updated on 12 September 2018) was used to search for data from the exosome samples. The most important searching parameters were set as the default settings: trypsin was selected as the enzyme, two missed cleavages were allowed for searching, the mass tolerance of the precursor was set to 10 ppm, and the product’s ions tolerance was 0.02 Da. Cysteine carbamidomethylation was specified as fixed modification, and methionine oxidation was chosen for variable modifications. The false discovery rate (FDR) < 1% was set for both peptide and protein identification. The data were filtered by the Q value, and “global normalization” was set as the “median” with enabled cross-run normalization.

### 4.5. Bioinformatics Analyses and Statistical Rationale

PANTHER GO-slim 14.0 was used to further perform the gene ontology analysis (GO), including the biological processes, cellular components, and molecular functions [59]. Pathway enrichment analysis was conducted by using the Kyoto Encyclopedia of Genes and Genomes database (KEGG), https://www.genome.jp/kegg. STRING 11.5 (https://cn.string-db.org/) enabled the protein–protein interaction network (PPI) analysis.

Statistical analysis was performed in IBM SPSS 26.0, in which normally distributed continuous variables were described as the mean ± standard error, and non-normally distributed continuous variables were described as the median and interquartile range. All *t*-tests were performed as two-sided and were unpaired in comparisons between two groups. Intergroup comparisons were made using ANOVA analysis, and significance values were corrected via Bonferroni correction. One-way ANOVA was followed by Dunnett’s multiple comparisons test. Receiver operating characteristic (ROC) analysis was performed using IBM SPSS 26.0. The visualization of the results was enabled by the use of GraphPad Prism 8.0.1 software. The heat map visualization of the clusters’ members was conducted with the “gplots” package in R 4.1.2. The least absolute shrinkage and selection operator (LASSO) logistic regression analysis was performed by using the “glmnet” package in R 4.1.2 based on K-fold cross-validation. Logistic regression was implemented using IBM SPSS 26.0 with the stepwise forward method of calculation, omnibus tests, and Hosmer–Lemeshow tests. Correlation analysis was carried out by using IBM SPSS 26.0, with a Wilson correlation analysis for normally distributed data a Spearman’s correlation analysis for non-normally distributed data.

## 5. Conclusions

We isolated urine exosomes based on ultracentrifugation combined with a 0.22 μm filtration membrane and screened 371 significantly different expressed urinary exosome proteins, which were mainly enriched in the immune pathway and metabolic pathway, some of which could reflect the level of kidney function and hyperglycemia management. The urinary exosome protein PHYHD1 was identified as a non-invasive biomarker to differentially diagnose DN and NDRD with optimal efficacy.

## Figures and Tables

**Table 1:** Demographic and clinical characteristics of the subjects.

	DN (n = 12)	NDRD (n = 15)	T2DM (n = 9)	HC (n = 12)
Age (years)	49.08 ± 3.21	51.29 ± 2.50	60.11 ± 3.09 ^a^	47.83 ± 1.90
Sex (male/female)	(6/6)	(12/3)	(4/5)	(8/4)
Serum albumin (g/L)	32.24 ± 2.75 ^ab^	31.16 ± 3.82 ^a^	42.43 ± 1.04	44.35 ± 1.06
HbA1c (%)	7.54 ± 0.53 ^a^	6.3 ± 0.27 ^b^	9.56 ± 0.62 ^a^	5.44 ± 0.069
Plasma glucose (mmol/L)	8.20 ± 1.63 ^a^	5.42 ± 0.29	10.33 ± 1.57 ^a^	5.07 ± 0.14
Urinary albumin (g/24 h)	2.42 ± 0.59 ^b^	1.77 ± 0.50 ^b^	0.04 ± 0.03	
Serum creatinine (μmol/L)	169.10 ± 16.73 ^ab^	107.53 ± 18.32 ^ab^	69.01 ± 7.01	64.77 ± 3.64
BUN (mmol/L)	10.08 ± 1.47 ^ab^	6.15 ± 0.66 ^a^	5.18 ± 0.58	4.38 ± 0.17
eGFR (mL min^−1^ 1.73 m^−2^)	32.23 ± 3.46 ^b^	68.41 ± 14.44	120.34 ± 10.96	

All data are presented as the mean ± standard deviation. ^a^ *p* < 0.05 vs. HC; ^b^ *p* < 0.05 vs. T2DM; ^c^ *p* < 0.05 vs. NDRD. HbA1c, glycated hemoglobin; BUN, blood urea nitrogen; eGFR, estimated glomerular filtration rate.

**Table 2:** The number of proteins significantly differentially expressed among groups.

	Number of Differential Proteins	Upregulated Proteins	Downregulated Proteins
HC vs. DN	1263	497	766
HC vs. NDRD	1177	508	669
HC vs. T2DM	632	462	170
T2DM vs. DN	1200	337	863
T2DM vs. NDRD	1260	347	913
NDRD vs. DN	557	156	401

DN, diabetic nephropathy; NDRD, non-diabetic renal disease; T2DM, Type 2 diabetes mellitus; HC, healthy controls.

**Table 3:** The 20 proteins with the largest log_2_ fold change (Q-value ˂ 0.05) between isolated urine exosomes of DN (n = 12) and NDRD (n = 15) patients. The upregulated and downregulated proteins that are most abundant are colored in orange and blue, respectively.

Protein Names	Protein Descriptions	DN vs. NDRD		
		Genes	AVG Log_2_ Ratio	Q-Value
PEPC	Gastricsin	PGC	5.45	0.01
PLCL1	Inactive phospholipase C-like protein 1	PLCL1	5.20	0.01
PIP	Prolactin-inducible protein	PIP	4.84	<0.01
SEMG2	Semenogelin-2	SEMG2	4.15	0.01
SEMG1	Semenogelin-1	SEMG1	3.39	<0.01
GTR14	Solute carrier family 2, facilitated glucose transporter member 14	SLC2A14	3.32	0.01
PLA1A	Phospholipase A1 member A	PLA1A	3.13	0.03
ILDR1	Immunoglobulin-like domain-containing receptor 1	ILDR1	2.65	0.03
CRCT1	Cysteine-rich C-terminal protein 1	CRCT1	2.35	<0.01
SIDT1	SID1 transmembrane family member 1	SIDT1	2.29	<0.01
PERM	Myeloperoxidase	MPO	−3.50	0.02
FIG4	Polyphosphoinositide phosphatase	FIG4	−3.38	<0.01
M4K4	Mitogen-activated protein kinase kinase 4	MAP4K4	−3.34	0.03
TETN	Tetranectin	CLEC3B	−3.26	0.02
LV746	Immunoglobulin lambda variable 7-46	IGLV7-46	−3.22	<0.01
PKP3	Plakophilin-3	PKP3	−3.13	0.02
GNTK	Probable glucokinase	IDNK	−3.09	0.04
H3PS2	Histone HIST2H3PS2	H3-2	−3.06	0.04
PERE	Eosinophil peroxidase	EPX	−3.04	0.02
SP100	Nuclear autoantigen Sp-100	SP100	−3.03	0.01

**Table 4:** Characteristics of the samples used in validation.

Variable	DN (n = 8)	NDRD (n = 5)	T2DM (n = 7)	HC (n = 9)
Age (years)	57.50 ± 7.71	58.00 ± 5.00	54.29 ± 11.64	47.67 ± 6.00
Sex (male/female)	(5/3)	(3/2)	(4/3)	(5/4)
Serum albumin (g/L)	34.20 ± 7.47 ^a^	34.92 ± 12.43	42.49 ± 4.20	45.03 ± 2.55
HbA1c (%)	7.39 ± 1.60 ^a^	6.62 ± 0.99	9.57 ± 2.32 ^a^	5.43 ± 0.24
Plasma glucose (mmol/L)	6.99 ± 2.21	6.50 ± 2.88	11.21 ± 5.24 ^a^	5.09 ± 0.36
Urinary albumin (g/24 h)	2.71 ± 1.98 ^ab^	2.83 ± 4.11 ^a^	0.02 ± 0.05	
Serum creatinine (μmol/L)	150.44 ± 53.27 ^ab^	114.40 ± 35.06	71.94 ± 23.20	65.89 ± 10.67
BUN (mmol/L)	9.51 ± 2.53 ^ab^	7.97 ± 1.89	4.90 ± 1.80	4.67 ± 0.96
eGFR (mL min^−1^ 1.73 m^−2^)	39.85 ± 17.76 ^b^	50.48 ± 17.91	97.55 ± 25.17	

All data are presented as the mean ± standard deviation. ^a^ *p* < 0.05 vs. HC; ^b^ *p* < 0.05 vs. T2DM; ^c^ *p* < 0.05 vs. NDRD. HbA1c, glycated hemoglobin; BUN, blood urea nitrogen; eGFR, estimated glomerular filtration rate.

## Data Availability

The data that support the findings of this study are available from the authors upon reasonable request.

## Supplementary Materials

The following supporting information can be downloaded at https://www.mdpi.com/article/10.3390/ijms241713560/s1.

## Abbreviations

ACN	Acetonitrile
ADA	American Diabetes Association
AMBP	Alpha-1-microglobulin/bikunin precursor
AUC	Area under the curve
DIA	Data-independent acquisition
DN	Diabetic nephropathy
DPP-4	Dipeptidyl peptidase-4
eGFR	Estimated glomerular filtration rate
ESRD	End-stage renal disease
FA	Formic acid
FDR	False discovery rate
FSGS	Focal segmental glomerulosclerosis
GO	Gene ontology
HC	Healthy control
IAM	Iodoacetamide
IgAN	IgA nephropathy
IL-6	Interleukin-6
ISEV	International Society for Extracellular Vesicles
KEGG	Kyoto Encyclopedia of Genes and Genomes
LASSO	Least absolute shrinkage and selection operator
MN	Membranous nephropathy
MS	Mass spectrometry
NDRD	Non-diabetic renal disease
NTA	Nanoparticle tracking analysis
PBS	Phosphate-buffered saline
PHYHD1	Phytanoyl-CoA dioxygenase domain containing 1
PLA	People’s Liberation Army
PPI	Protein–protein interaction networks
ROC	Receiver operating characteristic curve
T1DM	Type 1 diabetes mellitus
T2DM	Type 2 diabetes mellitus
TEM	Transmission electron microscope
TGF-β	Transforming growth factor-β
UACR	Urinary albumin-to-creatinine ratio
