# Supplementary material for: Identification of a Non-Invasive Urinary Exosomal Biomarker for Diabetic Nephropathy Using Data-Independent Acquisition Proteomics
Source: Int J Mol Sci. 2023 Sep 1;24(17):13560. doi: 10.3390/ijms241713560 (PMC10488032; doi:10.3390/ijms241713560)

Exosome extraction and purification:

First, before renal biopsy conducted, the midstream of the first morning urine samples (of 50 mL each) were obtained from each eligible participant. Samples without protease inhibitors cocktails were immediately centrifuged (3803 g at 4◦C for 45 min; TX-400, Thermo Fisher Scientific) to remove cellular debris and organelles. The obtained supernatants were stored at -80◦C for exosome isolation. After sample collection phrase, all samples were thawed at 25◦C and centrifugated (3803 *g* at 4 ◦C for 45 min; TX-400, Thermo Fisher Scientific), after that the obtained supernatants were filtrated by a 0.22 µm polyethersulfone filter sterilization device (Steriflip® Vacuum Driven Sterile Filter, Millipore). The flow-through were centrifugated (200,000 g at 4◦C for 2h; Beckman Coulter, Optima XPN-100 with the 45-Ti rotor) to obtain the sedimental exosomal-containing pellets. The pellets were resuspended in 50 mL phosphate-buffered saline (PBS), and centrifugated (200,000 *g* at 4◦C for 2h; Beckman Coulter, Optima XPN-100 with the 45-Ti rotor) to wash the sedimental exosomal-containing pellets again. Then the pellets were resuspended in 1 mL PBS and concreated by repeated centrifugations (200,000 g at 4◦C for 2h; Beckman Coulter, Optima MAX-XP with the TLA 100.3 rotor). The final pellets of urine exosomes were resuspended in 60µL PBS and stored at -80◦C (Ultra Low Freezer; Thermo Scientific) until characterization and proteomic analysis.

Supplementary Figure S1:

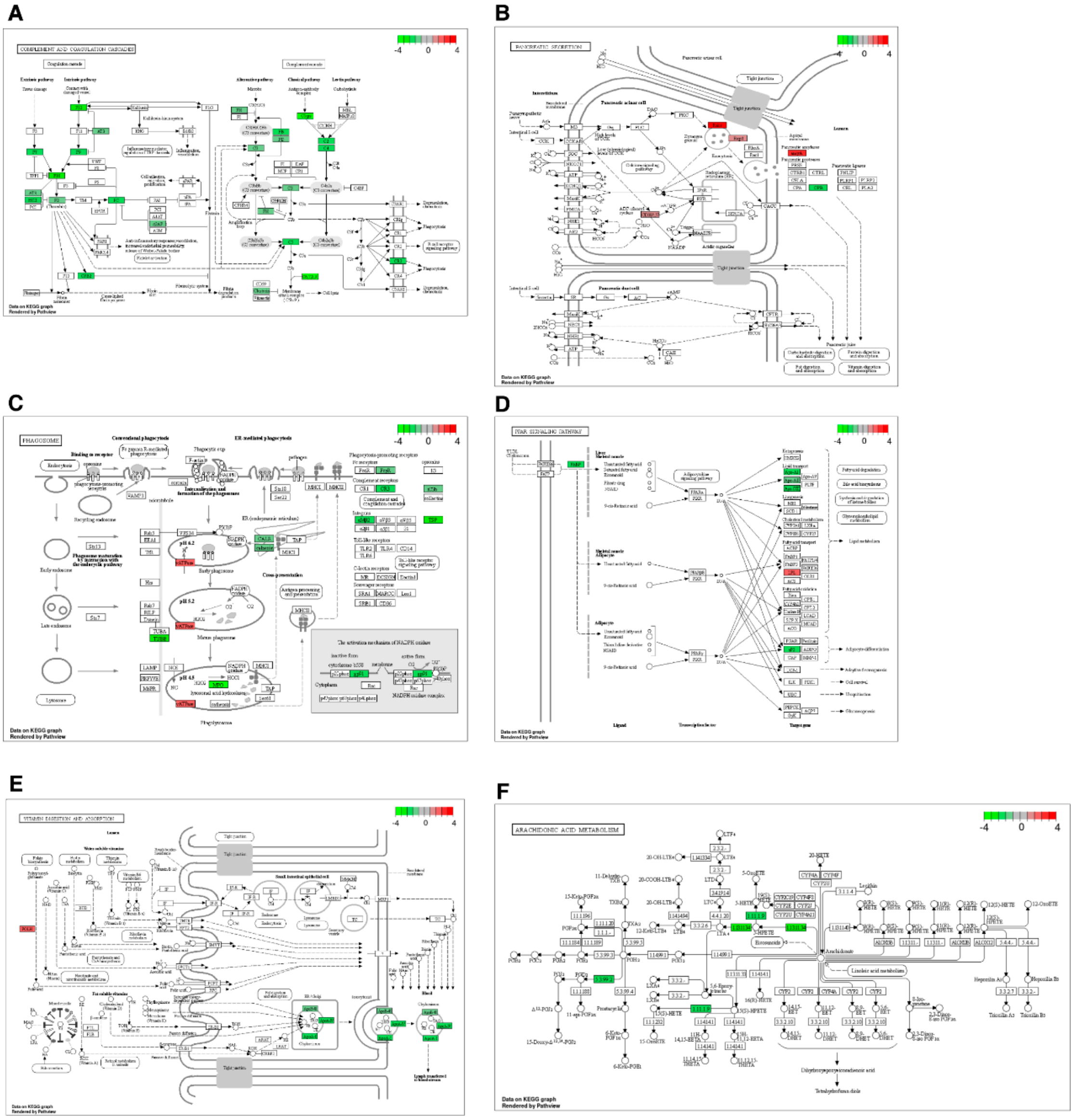

Supplement: Supplementary file 1 [file ijms-24-13560-s001.zip › Supplementary materials.pdf]
