# Supplementary material for: Identification of a Non-Invasive Urinary Exosomal Biomarker for Diabetic Nephropathy Using Data-Independent Acquisition Proteomics
Source: Int J Mol Sci. 2023 Sep 1;24(17):13560. doi: 10.3390/ijms241713560 (PMC10488032; doi:10.3390/ijms241713560)
Supplement: Supplementary file 1 [file ijms-24-13560-s001.zip › Supplementary Table S2.pdf]

Supplemental Table S2. ROC analysis of 10 potential candidates of urine exosome proteins to differential diagnose DN and NDRD patients

ROC analysis

Variants	AUC	Standard Error	Significance	95% CI	
				Lower limits	Upper limits
PHYHD1	0.919444	0.058597	0.00023	0.804597	1
APOB	0.877778	0.072291	0.000906	0.73609	1
C12orf4	0.827778	0.081215	0.003991	0.6686	0.986956
MFSD10	0.813889	0.08917	0.005835	0.63912	0.988658
COMP	0.8	0.0877	0.008415	0.62811	0.97189
SIDT1	0.777778	0.095284	0.014697	0.591025	0.964531
C18orf63	0.744444	0.102439	0.031795	0.543667	0.945222
BPIFB1	0.727778	0.100859	0.045436	0.530098	0.925457
RETN	0.722222	0.098547	0.050962	0.529073	0.915372
PPP1R12A	0.575	0.117533	0.510068	0.344639	0.805361
