# Supplementary material for: Identification of a Non-Invasive Urinary Exosomal Biomarker for Diabetic Nephropathy Using Data-Independent Acquisition Proteomics
Source: Int J Mol Sci. 2023 Sep 1;24(17):13560. doi: 10.3390/ijms241713560 (PMC10488032; doi:10.3390/ijms241713560)
Supplement: Supplementary file 1 [file ijms-24-13560-s001.zip › Supplementary Table S3.pdf]

Supplemental Table S3. Univariate logistic regression analysis of 10 potential candidates

Protein name	β value	OR 95%CI	P value
APOB	-.069	0.933（0.883, 0.987）	0.015*
BPIFB1	0.332	1.394（0.906, 2.144）	0.131
C12orf4	3.810	45.164（1.123, 1816.743）	0.043*
COMP	-0.416	0.660（0.459, 0.949）	0.025*
C18orf63	-0.054	0.948（0.896, 1.003）	0.062
MFSD10	-0.408	0.665（0.438, 1.009）	0.550
PPP1R12A	0.223	NA	0.565
PHYHD1	2.451	11.604（1.830, 73.574）	0.009*
RETN	-0.008	0.992（0.978, 1.007）	0.291
SIDT1	-0.069	0.933（0.883, 0.987）	0.015*

The significance is marked with *（$p < 0.05$）
